# Water-Resistant Poly(ethylene oxide) Electrospun Membranes Enabled by In Situ UV-Cross-Linking for Efficient Daytime Radiative Cooling

**DOI:** 10.3390/molecules30020421

**Published:** 2025-01-20

**Authors:** Haiyan Zhang, Qingpeng Wang, Zhiguang Xu, Yan Zhao

**Affiliations:** 1 College of Textile and Clothing Engineering, Soochow University, Suzhou 215123, China; 20204015004@stu.suda.edu.cn (H.Z.);; 2 China-Australia Institute for Advanced Materials and Manufacturing, College of Biological, Chemical Sciences and Engineering, Jiaxing University, Jiaxing 314001, China

**Keywords:** electrospun membranes, poly(ethylene oxide), radiative cooling, UV-cross-linking, water resistance

## Abstract

Daytime radiative cooling, based on selective infrared emissions through atmospheric transparency windows to outer space and the reflection of solar irradiance, is a zero-energy and environmentally friendly cooling technology. Poly(ethylene oxide) (PEO) electrospun membranes have both selective mid-infrared emissions and effective sunlight reflection, inducing excellent daytime radiative cooling performance. However, PEO is highly water soluble, which makes electrospun PEO membranes unable to cope with rainy conditions when used for outdoor daytime radiative cooling. Herein, we report an in situ UV-crosslinking strategy for preparing PEO electrospun membranes with water resistance for the application of daytime radiative cooling. Acrylate-terminated PEO was synthesized and mixed together with cross-linking agents and photoinitiators to prepare the electrospinning solution. During electrospinning, the nanofibers were irradiated with UV light to initiate the cross-linking. For a membrane with a thickness of 200 μm, the average solar reflectance was 89.6%, and the infrared emissivity (8–13 μm) was 96.3%. Although slight swelling happens to the cross-linked membrane once it comes into contact with water, the fibrous morphology shows no obvious change when prolonging the water soaking time, indicating excellent water resistance. The outdoor cooling performance test results showed that compared to the average temperature of the air in the test box, the average temperature drop in the membrane before and after water soaking was 13.8 °C and 11.5 °C, respectively. Crosslinked PEO-based electrospun membranes with both water resistance and radiative cooling performance may have real applications for outdoor daytime radiative cooling.

## 1. Introduction

Radiative cooling materials reflect solar irradiance and radiate heat through the “atmospheric transparency window” (8–13 μm) to achieve a cooling effect [1,2,3]. Unlike traditional electricity-based cooling technologies that consume tremendous amounts of energy and produce substantial pollution, daytime radiative cooling is a zero-energy and environmentally friendly cooling strategy. To achieve high cooling performance, daytime radiative cooling materials should have high infrared emissivity and minimized solar absorption [4,5,6]. With the rapid development of the materials and nanotechnology fields, diverse radiative cooling materials with strong emissivity and reflectivity are gradually appearing, such as multilayer structures [7,8], photonic crystal structure [9], nanoparticle doped materials [10,11,12], coating metamaterials [13,14,15], and biomimetic materials [10,16,17,18].

Electrospinning is a technique applicable to almost all polymers and can produce continuous ultra-fine fibers with diameters ranging from several micrometers to hundreds of nanometers [19]. In particular, the characteristics of electrospun membranes, including fiber diameter, morphology, alignment, porosity, and surface area, can be easily adjusted by optimizing the parameters of electrospinning, such as the electric field, solvent types, and spinning conditions. Since the sizes of electrospun fibers are comparable to the wavelength range of sunlight, electrospun membranes with disordered fiber arrangement structures have relatively high sunlight reflectivity [20,21,22,23]. Thus, electrospinning is an efficient nanofabrication method for preparing daytime radiative cooling materials using polymers with high infrared emissivity in the atmospheric window, including polymers with broadband emissions covering the whole mid-infrared wavelength, such as cellulose acetate (CA) [24] and polylactic acid (PLA) [25], and polymers with selective emissions covering the atmospheric window, such as polyethylene oxide (PEO) [26] and polyvinylidene fluoride (PVDF) [10,12,27].

Among these polymers, PEO only has C-O, C-C, and C-H bonds. C-O stretching results in strong IR absorption at 7.7–10 μm, while C-C and C-H bonds just have narrow absorption peaks centered at the wavelengths of 3.4, 3.5, 6.8, 7.3, and 13.7 μm [28], thus resulting in PEO with a desirable selective absorption band that overlaps with the 8–13 μm atmospheric transparency window and no obvious absorption outside of this region (i.e., highly selective mid-infrared thermal emission) [26,29]. Therefore, PEO electrospun membranes that have selective mid-infrared emission and effective scattering nanostructures have been prepared for the application of daytime radiative cooling. For instance, Zhu’s group fabricated PEO electrospun membranes containing randomly stacked nanofibers (diameter size centered at ~800 nm) for radiative cooling via a scalable roll-to-roll electrospinning method. The PEO nanofibrous membranes showed a high reflectivity of 96.3% in the sunlight region (0.3–2.5 μm) and a high emissivity of 78% in the wavelength region of 8–13 μm, thereby resulting in ~5 °C sub-ambient cooling under sunlight. Later, to improve the mechanical properties and UV stability of PEO electrospun membranes, they doped potassium titanate (K_2_Ti_6_O_13_) nanofibers into the PEO electrospun fibers, and they observed enhancements in the strength, elongation at break, Young’s modulus, and UV stability by 2.3, 1.6, 7, and 12 times, respectively [30]. However, as daytime radiative cooling materials are used outdoors, the intrinsic water-solubility of PEO results in the PEO electrospun membranes’ inability to cope with rainy conditions, significantly restricting their practical applications.

Although the cross-linking of PEO can be realized via radiation techniques (electron beam [31], gamma-ray [32], or UV light [33]) or chemical cross-linking through the reaction between the end groups in PEO with cross-linking agents [34], the physicochemical properties and biocompatibility of cross-linked PEO make it mostly used for biomedical applications such as drug delivery [35] and cell culture scaffolds [36]. Regarding electrospinning, PEO is mainly used as an assistant polymer because of its good electrospinnability, and it is used to improve the spinnability and facilitate the fiber formation of hardly electrospinnable or even non-electrospinnable polymers such as chitosan [37], collagen [38], and alginate [39]. The PEO in the composite nanofibers can be cross-linked to form a relatively stable part of the nanofibers [40], but in most cases, PEO is also employed as a sacrificial template, and it is extracted by soaking nanofibers in water to leave only the main component of nanofibers. In this way, the as-generated porous structure in the nanofibers can be utilized for performance improvement in applications such as dye adsorption (due to increased surface area) [37] and radiative cooling (due to increased light scattering). So far, research works on the cross-linking of single-component PEO electrospun nanofibers are extremely rare [33,34], and the fiber morphology’s stability over water soaking has not been explored. Therefore, studies on the cross-linking of PEO electrospun nanofibers used for radiative cooling and the effects of the water swelling of cross-linked nanofibers on optical properties are highly desired in order to use PEO electrospun membranes in real applications with respect to daytime radiative cooling.

In this work, an in situ UV-cross-linking strategy was developed for preparing electrospun PEO membranes with water resistance for the application of daytime radiative cooling. The hydroxyl group at both ends of PEO was first reacted with ethyl isocyanate acrylate (ISA) to obtain PEO-ISA-containing acrylate groups at both ends, and then, the PEO-ISA spinning solution was prepared with the addition of a cross-linking agent, trimethylolpropane triacrylate (TMPTA), and a photoinitiator, benzophenone. During electrospinning, the nanofibers were irradiated with UV light (320 nm at 70 mW/cm^2^) to initiate cross-linking. To further improve sunlight reflection, Al_2_O_3_ particles (diameter 30 nm) were doped into the fibers. In contrast to the uncross-linked PEO electrospun membrane that completely disappeared once immersed in water, the cross-linked membrane could still maintain its fibrous morphology even after soaking in water for 1 h. The optical properties and outdoor radiative cooling performance of the cross-linked membrane before and after water soaking were further tested. We hope that such a strategy for in situ UV-cross-linking may promote the real application of PEO electrospun membranes for outdoor daytime radiative cooling.

## 2. Results and Discussion

**Figure 1:**
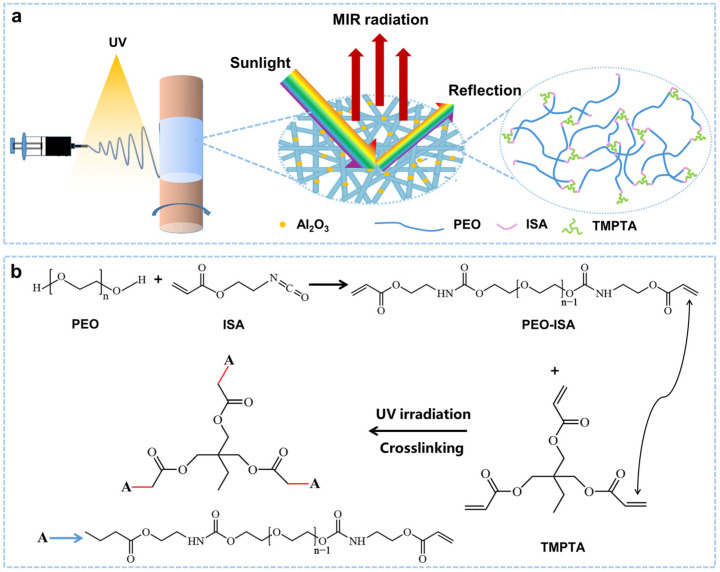
(**a**) Schematic illustrating the preparation process for water-resistant PEO-based electrospun membranes. (**b**) Reactions involved in the synthesis of PEO-ISA and in situ UV-cross-linking.

As shown in Figure 1, an in situ UV-cross-linking method was developed to prepare PEO-based electrospun membranes with water resistance for outdoor daytime radiative cooling. The hydroxyl groups at both ends of PEO were first reacted with ISA to obtain PEO-ISA, which contains acrylate groups at both ends, and then, the PEO-ISA spinning solution was prepared with the addition of a cross-linking agent (TMPTA) and a photoinitiator (benzophenone). During electrospinning, UV light (320 nm at 70 mW/cm^2^) was used to irradiate the nanofibers to initiate cross-linking. In situ UV-cross-linking allows the formation of a cross-linked stable molecular network (Figure 1a). The mechanism for the in situ UV-cross-linking of PEO-ISA with TMPTA is presented in Figure 1b. Al_2_O_3_ particles (diameter 30 nm) were doped into the nanofibers to improve sunlight reflection.

**Figure 2:**
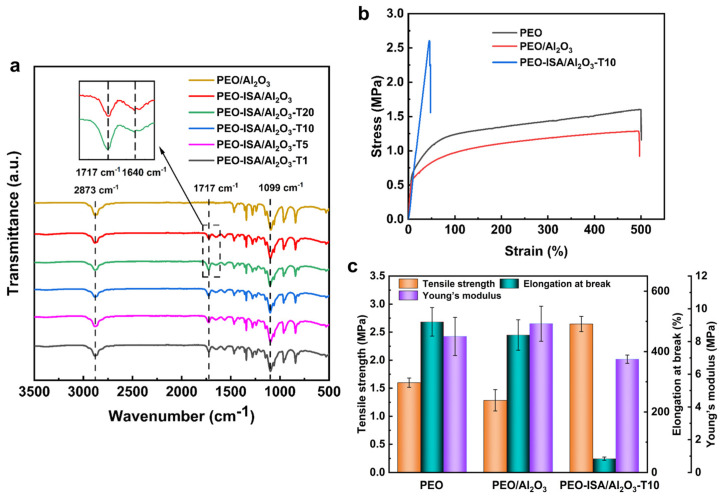
(**a**) FTIR spectra of PEO-based electrospun membranes. (**b**) Tensile stress–strain curves and (**c**) the corresponding values of tensile strength, elongation at break, and Young’s modulus of PEO-based electrospun membranes.

Figure 2a shows the FTIR spectra of electrospun nanofibrous membranes. All these PEO-based membranes showed the typical absorption peaks of PEO at 1099 cm^−1^ and 2873 cm^−1^, which correspond to C-O-C and C-H bond vibrations. Compared to the spectrum of PEO/Al_2_O_3_ membranes, for the membranes spun with PEO-ISA, there appeared characteristic peaks at 1640 cm^−1^ and 1717 cm^−1^, corresponding to the stretching vibrations of C=C and C=O, respectively. Moreover, as shown in the inset of Figure 2a, the intensity of the peak 1717 cm^−1^ was higher for the membrane cross-linked with TMPTA due to the existence of C=O groups in TMPTA molecules.

The mechanical properties of PEO-based membranes were investigated via tensile tests. As shown in Figure 2b,c, compared to the uncross-linked PEO and PEO/Al_2_O_3_ electrospun membranes, the tensile strength of the cross-linked PEO-ISA/Al_2_O_3_-T10 membrane significantly increased due to the cross-linking that creates covalent bonding among the molecular chains of PEO. The tensile strength at the break for the PEO-ISA/Al_2_O_3_-T10 membrane was 2.65 MPa, which increased by about 60% and nearly 100% compared to that of the PEO and PEO/Al_2_O_3_ membranes. For the cross-linked PEO-ISA/Al_2_O_3_-T10 membrane, the elongation at break was just 45.7%, which is much lower than that of uncross-linked PEO and PEO/Al_2_O_3_ membranes due to the cross-linked network that resists deformation under stress. The Young’s modulus was slightly increased with the addition of Al_2_O_3_, while the cross-linked PEO-ISA/Al_2_O_3_-T10 membrane showed a lowered modulus. This phenomenon was also observed in a previous report that used TMPTA as a cross-linker for PEO [33].

**Figure 3:**
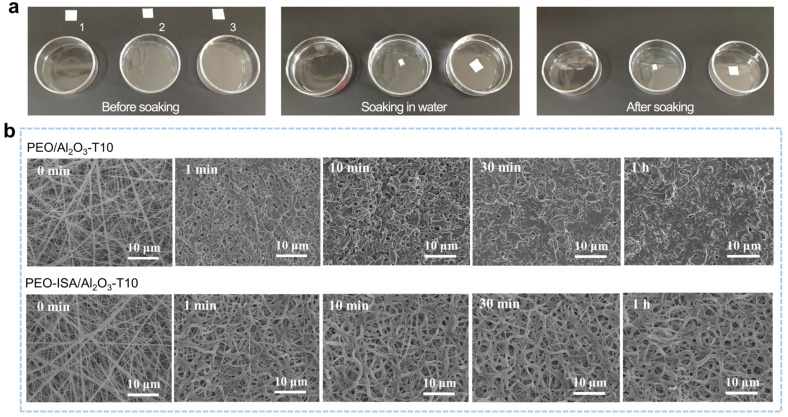
(**a**) Photos showing changes in the PEO (1), PEO/Al_2_O_3_-T10 (2), and PEO-ISA/Al_2_O_3_-T10 (3) electrospun membranes before and after soaking in water for 30 min. (**b**) SEM images of PEO/Al_2_O_3_-T10 and PEO-ISA/Al_2_O_3_-T10 after soaking in water for 1 min, 10 min, 30 min, and 1 h, respectively.

The water resistance of PEO-based electrospun membranes was tested by soaking the membranes in water for a certain time period. As shown in Figure 3a, the PEO electrospun membrane completely dissolved in water within 10 min. The sample of PEO/Al_2_O_3_-T10 (without the end-capping with ISA) partially dissolved in water and experienced size shrinkage. In this case, the weak water resistance can be attributed to the photo-induced hydrogen abstraction reactions from PEO and the subsequent cross-linking with TMPTA [33]. In contrast, the PEO/Al_2_O_3_-T10 sample maintained its integrity in water, indicating that the in situ UV cross-linking is effective for imparting PEO-based electrospun membranes with water resistance.

Figure 3b shows the SEM images of the PEO/Al_2_O_3_-T10 and PEO-ISA/Al_2_O_3_-T10 membranes before and after soaking in water for different time periods followed by being naturally dried. It can be seen that the fibrous morphology of PEO/Al_2_O_3_-T10 became hard to identify after being soaked in water for just 1 min. With an increase in the water soaking time, the pores between the electrospun fibers tended to disappear, and film-like surface morphology was observed after 1 h of water soaking. However, for the PEO-ISA/Al_2_O_3_-T10 membrane, the fibrous morphology was maintained even after 1 h of water soaking, though the fibers became curly due to the slight swelling in water. In particular, the ultrafine fibers still existed, indicating the high cross-linking efficiency of the in situ UV cross-linking method proposed in this work. More importantly, compared to the fibrous morphology after 1 min of water soaking, the change in the fibrous morphology was not obvious when prolonging the duration of water soaking. Furthermore, the PEO-ISA/Al2O3-T10 membrane was subjected to repeated water soaking–drying processes. It was found that there was no further change in the fibrous morphology after the first water soaking–drying cycle (Figure S1). These results indicate that the PEO-ISA/Al_2_O_3_-T10 membrane has excellent water resistance and can provide reliable functions even after encounters with rainy weather.

**Figure 4:**
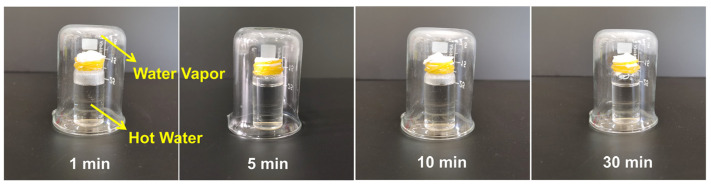
Photographs showing the moisture permeability of PEO-ISA/Al_2_O_3_-T10 after different water soaking time periods.

To visually confirm that the cross-linked PEO-ISA/Al_2_O_3_-T10 membrane is still moisture-permeable after water soaking based on its preserved fibrous morphology, a simple experiment was designed. As shown in Figure 4, the sample of the PEO-ISA/Al_2_O_3_-T10 membrane after soaking in water for different time periods was tightly sealed at the opening of a small glass bottle containing hot water, and a glass beaker was used to cover the small glass bottle. It can be observed that the moisture vapor coming from the hot water can penetrate through the electrospun membranes and condense on the inner wall of the glass beaker, confirming the fibrous morphology of the water-soaked membranes and the as-resulted moisture permeability.

**Figure 5:**
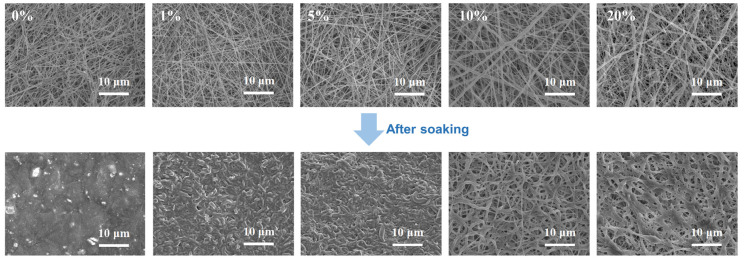
SEM images of PEO-based electrospun membranes prepared with different TMPTA mass fractions of 0%, 1%, 5%, 10%, and 20%, respectively, before and after soaking in water for 30 min.

The influence of the amount of cross-linker TMPTA on the fiber morphology and water resistance was investigated by observing the microscopic morphologies of the membranes prepared with different mass fractions of TMPTA (relative to PEO-ISA) before and after soaking in water for 30 min. As shown in Figure 5, for membranes without TMPTA (PEO-ISA/Al_2_O_3_-T0) or with relatively low amounts of TMPTA (PEO-ISA/Al_2_O_3_-T1 and PEO-ISA/Al_2_O_3_-T5), fibrous morphology was destroyed once soaked in water. Water resistance was achieved for both samples of PEO-ISA/Al_2_O_3_-T10 and PEO-ISA/Al_2_O_3_-T20, while too high contents of TMPTA resulted in the appearance of beads on the fibers. Therefore, PEO-ISA/Al_2_O_3_-T10 was considered optimal.

**Figure 6:**
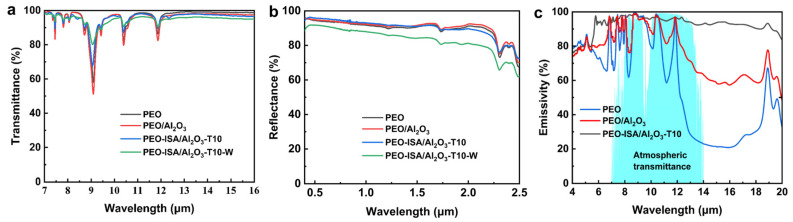
Optical properties of PEO-based electrospun membranes: (**a**) MIR transmittance, (**b**) solar reflectance, and (**c**) MIR emissivity spectra.

Through the in situ UV-cross-linking, we successfully imparted PEO-based nanofiber membranes with water resistance. Although the fibrous morphology of the nanofibers was preserved, the nanofibers changed from straight to curly after soaking in water. As radiative cooling materials, the optical properties of electrospun membranes are the primary factors affecting the cooling performance. In the following, the optical properties and cooling performance of different nanofiber membranes with a thickness of 200 μm were tested. As shown in Figure 6a, in the “atmospheric window” range of 8–13 μm, the average transmittance of PEO, PEO/Al_2_O_3_, PEO-ISA/Al_2_O_3_-T10, and PEO-ISA/Al_2_O_3_-T10-W (i.e., the PEO-ISA/Al_2_O_3_-T10 sample after 30 min water soaking) was 93.6%, 92.3%, 93.7%, and 93.4%, respectively. It can be observed that UV cross-linking has little effect on the average mid-infrared transmittance of electrospun membranes. The solar reflectance of PEO, PEO/Al_2_O_3_, and PEO-ISA/Al_2_O_3_-T10 in the range of 0.28–2.5 μm was 90.2%, 91.1%, and 89.6%, respectively (Figure 6b). After water soaking, the solar reflectance of PEO-ISA/Al_2_O_3_-T10 was reduced to 82.7% due to the swelling and deformation of nanofibers. Figure 6c shows the infrared emission spectra of the electrospun membranes. In the atmospheric window range of 8–13 μm, the average infrared emissivity of PEO, PEO/Al_2_O_3_, and PEO-ISA/Al_2_O_3_-T10 was 75.8%, 90.0%, and 96.3%, respectively. The enhanced infrared emission of the cross-linked membrane might be attributed to the vibrations of the introduced C=C and C=O groups.

**Figure 7:**
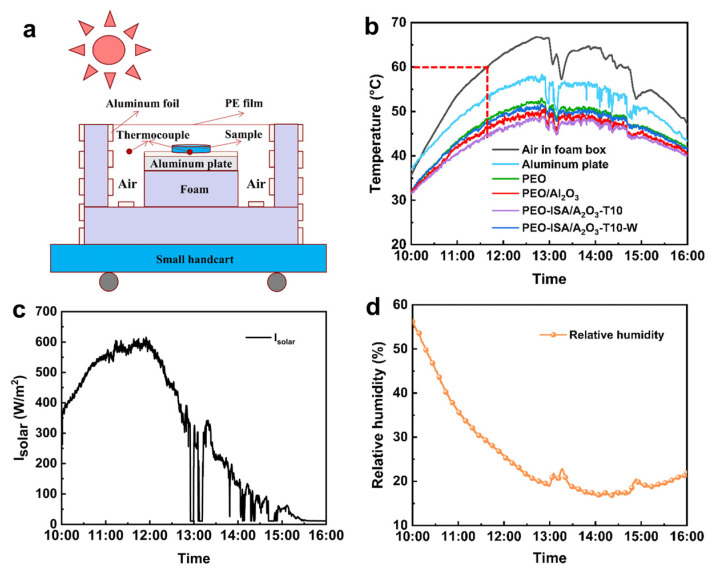
(**a**) Schematic diagram of the test setup. (**b**) Outdoor radiative cooling test results for the PEO-based electrospun membranes. Changes in (**c**) solar power density and (**d**) ambient relative humidity over time during the test process.

The outdoor radiative cooling performance of PEO-based electrospun membranes was tested using a homemade setup (Figure 7a and Figure S2). The test was conducted in a polystyrene foam box to eliminate the effect of heat conduction. Infrared transparent PE film (10 μm thick) was used to cover the top opening of the foam box to prevent heat convection. The foam box was covered with aluminum foil to reflect the surrounding thermal radiation. A thermocouple was placed under the sample to record the real-time temperature change. The test results are shown in Figure 7b, and the variations in solar irradiance and environmental relative humidity over time during the test process are shown in Figure 7c and Figure 7d, respectively. It can be seen that the PEO-ISA/Al_2_O_3_-T10 membrane exhibited excellent daytime radiative cooling performance, significantly outperforming the PEO and PEO/Al_2_O_3_ membranes. When the average temperature of the air in the foam box was 57.2 °C, the average temperatures of the PEO, PEO/Al_2_O_3_, and PEO-ISA/Al_2_O_3_-T10 fiber membranes were 46.5 °C, 44.8 °C, and 43.7 °C, respectively. When the temperature of the air in the foam box reached 60.0 °C, the temperature reduction in the PEO-ISA/Al_2_O_3_-T10 membrane compared to the aluminum plate, PEO membrane, and PEO/Al_2_O_3_ membrane was 8.3 °C, 3.3 °C, and 1.4 °C, respectively. The better outdoor cooling performance of the PEO-ISA/Al_2_O_3_-T10 membrane than that of the PEO/Al_2_O_3_ membrane can be attributed to its higher infrared emissivity. After water soaking, the outdoor radiative cooling capability of the cross-linked membrane decreased due to the reduced sunlight reflectance as described above, but radiative cooling performance still outperformed that of the uncross-linked PEO electrospun membrane. Compared to the average temperature of the air in the foam box, the average temperature drop in the PEO-ISA/Al_2_O_3_-T10 membrane before and after water soaking was 13.8 °C and 11.5 °C, respectively. These results indicate that the in situ cross-linking strategy ensures the water resistance of PEO-based electrospun membranes and, meanwhile, the daytime radiative cooling performance for practical applications. Here, it should be pointed out that the cooling performance is compared to the temperature of the air in the enclosed testing box rather than the true ambient temperature. To obtain a cooling effect under realistic conditions, it is crucial to further enhance the solar reflectivity of the membranes, as a net sub-ambient cooling effect during daytime hours typically requires a solar reflectivity of around 90% or above [41]. In addition, the durability against long-term UV irradiation from sunlight is also important for practical applications. To improve UV stability, inorganic nanomaterials that can absorb high-energy UV photons and transform them into less harmful heat can be doped into the fibers to prevent degradation [30,42].

## 3. Experimental Section

### 3.1. Materials

Poly(ethylene oxide) (PEO, Mw = 800,000) was purchased from Hefei BASF Biotechnology Co., Ltd. (Hefei, China). Nanoalumina (Al_2_O_3_, diameter 30 nm), and benzophenone was purchased from Shanghai Maclin Biochemical Technology Co., Ltd. (Shanghai, China). Acetonitrile and trimethylolpropane triacrylate (TMPTA) were supplied by Shanghai Aladdin Biochemical Technology Co., Ltd. (Shanghai, China). Isocyanate ethyl acrylate (ISA) was purchased from Shanghai Titan Technology Co., Ltd. (Shanghai, China). Acetone was purchased from Yonghua Chemical Technology (Jiangsu) Co., Ltd. (Suzhou, China). Dichloromethane was purchased from Jiangsu Qiangsheng Functional Chemical Co., Ltd. (Suzhou, China). 

### 3.2. Preparation of Acrylate-Terminated PEO (PEO-ISA)

Acrylate-terminated PEO was synthesized via end-capping the end –OH groups of PEO using ISA according to the method reported in our previous work [43,44]. Typically, 5 g of PEO was added to 100 mL of dichloromethane, and then, 0.90 g of ISA was added slowly dropwise into the stirred PEO solution, followed by continuous stirring at room temperature for 12 h. The product was poured into a glass Petri dish and dried to obtain PEO-ISA.

### 3.3. Preparation of Electrospinning Solutions

The PEO-ISA/Al_2_O_3_ solution was prepared by adding 1 g of PEO-ISA and 0.04 g of Al_2_O_3_ into acetonitrile and acetone (1:1) and stirring for 4 h at 30 °C. Then, the solution was ultrasonically treated for 30 min to improve the dispersion of Al_2_O_3_ particles. TMPTA relative to the PEO-ISA mass fraction of 1%, 5%, 10%, or 20% was added into the solution under light-proof conditions, and the solution was stirred for 30 min at room temperature. The photoinitiator benzophenone with a mass percentage of 1% relative to PEO-ISA was added to the solution to prepare a homogeneous PEO-ISA/Al_2_O_3_/TMPTA spinning solution. The as-prepared electrospun membrane was named PEO-ISA/Al_2_O_3_-T, and according to the mass fraction of TMPTA relative to PEO-ISA, five samples (PEO-ISA/Al_2_O_3_-T1, PEO-ISA/Al_2_O_3_-T5, PEO-ISA/Al_2_O_3_-T10, and PEO-ISA/Al_2_O_3_-T20) were obtained. A control sample of PEO-ISA/Al_2_O_3_- was prepared using the PEO-ISA/Al_2_O_3_ solution without the addition of TMPTA.

To study the effect of the concentration of PEO solutions on fiber morphology and thus solar reflectance, the PEO concentration was set to 3 wt%, 4 wt%, 5 wt%, 6 wt%, and 7 wt%, respectively, to obtain samples of PEO-3, PEO-4, PEO-5, PEO-6, and PEO-7. Test results showed that PEO-5 is optimal based on the fiber morphology and reflectivity of the membranes (Figures S3 and S4), and thus, PEO-5 was used as a control sample (abbreviated as PEO) to reveal the poor water resistance of uncross-linked PEO nanofibers.

The effect of Al_2_O_3_ content was studied by varying the mass fraction of Al_2_O_3_ relative to PEO from 2%, 4%, 6%, to 8% to obtain samples of PEO/Al_2_O_3_-2, PEO/Al_2_O_3_-4, PEO/Al_2_O_3_-6, and PEO/Al_2_O_3_-8. Test results showed that PEO/Al_2_O_3_-4 is optimal based on the reflectivity of the membranes (Figure S5), which was then also used as a control sample (abbreviated as PEO/Al_2_O_3_) for the cross-linked PEO-ISA/Al_2_O_3_-T10 (the mass fraction of Al_2_O_3_ relative to PEO-ISA is also 4%).

Moreover, PEO/Al_2_O_3_-T10 was prepared as a control sample using pristine PEO instead of PEO-ISA for the sample of PEO-ISA/Al_2_O_3_-T10 to show the necessary end-capping of PEO with ISA.

### 3.4. Preparation of Electrospun Membranes

For electrospinning, the spinning solution was placed in a 20 mL syringe. For solutions containing TMPTA, the syringe was covered to protect the solution from light irradiation, and UV light (320 nm, intensity 70 mW/cm^2^) was used to irradiate the nanofibers to initiate UV cross-linking during the electrospinning process. Spinning conditions were 25 °C and 55% relative humidity. The spinning voltage was 12 kV, the receiving distance was 15 cm, the roller speed was 800 r/min, and the flow rate was 3 mL/h. After electrospinning, the as-prepared electrospun membranes (thickness 200 μm) were dried in an oven at 50 °C for 1 h.

### 3.5. Characterizations

A scanning electron microscope (Hitachi Regulus 8230, Hitachi, Tokyo, Japan) was used to observe the microscopic morphology of the samples. A Fourier transform infrared (FTIR) spectrometer (Vertex 70-Hyperion 2000, Bruker, Billerica, MA, USA) with an integrating sphere attachment was used to measure the mid-infrared transmittance of fibrous membrane samples. A UV-VIS-NIR spectrophotometer (Hitachi UH4150, Hitachi, Tokyo, Japan) with an integrating sphere attachment was used to measure the reflectance of the fibrous membrane samples. The infrared emissivity of the samples was measured using a Nicolet iS50 FTIR spectrometer (Thermo Fisher Scientific, Waltham, MA, USA) equipped with an integrating sphere attachment. The mechanical properties of the samples (1 cm × 4 cm) were tested using a Universal Material Testing Machine (UMTM) type 3365 (Instron, Norwood, MA, USA). The distance between the upper and lower grips of the universal material testing machine was set at 20 mm, and the tensile rate was set at 20 mm/min. For each sample, the test was conducted three times (Figure S6).

## 4. Conclusions

An in situ UV cross-linking strategy was developed based on the synthesis of PEO with acrylate terminated at both ends for preparing water-resistant PEO-based electrospun membranes for the application of daytime radiative cooling. Although slight swelling happens to the cross-linked membrane once it is in contact with water, the fibrous morphology shows no obvious changes when prolonging water soaking times, implying excellent water resistance that can meet the challenge of rainy conditions. The outdoor cooling performance test results showed that compared to the average temperature of the air in the outdoor cooling test box, the average temperature drop in the membrane before and after water soaking was 13.8 °C and 11.5 °C, respectively. We anticipate that this in situ cross-linking strategy that assures both water resistance and radiative cooling performance may lead to the real application of PEO-based electrospun membranes in the field of daytime radiative cooling.

## Data Availability

The original contributions presented in this study are included in the article/Supplementary Material. Further inquiries can be directed to the corresponding authors.

## Supplementary Materials

The following supporting information can be downloaded at: https://www.mdpi.com/article/10.3390/molecules30020421/s1. Figure S1. SEM images of PEO-ISA/Al_2_O_3_-T10 after (a) the second and (b) the third water soaking-drying cycle (water soaking time is 1 h). Figure S2. Digital photos of the test setup using for outdoor cooling performance test: with samples (left), and without samples (right, showing the position of the thermocouples). Figure S3. SEM images of PEO electrospun membranes prepared with spinning solutions having different concentrations: (a) 3 wt%; (b) 4 wt%; (c) 5 wt%; (d) 6 wt%; (e) 7 wt%. Figure S4. Reflectance of PEO electrospun membranes prepared with spinning solutions having different concentrations. Figure S5. Reflectance of PEO/Al_2_O_3_ electrospun membranes prepared with different Al_2_O_3_ mass fractions. Figure S6. Tensile stress-strain curves for the PEO, PEO/Al_2_O_3_, and PEO-ISA/Al_2_O_3_-T10 electrospun membranes.

## References

[1] Chen M., Pang D., Chen X., Yan H., Yang Y. (2021). Passive daytime radiative cooling: Fundamentals, material designs, and applications. Ecomat.

[2] Kecebas M.A., Menguc M.P., Kosar A., Sendur K. (2017). Passive radiative cooling design with broadband optical thin-film filters. J. Quant. Spectrosc. Radiat. Transf..

[3] Yu X., Chan J., Chen C. (2021). Review of radiative cooling materials: Performance evaluation and design approaches. Nano Energy.

[4] Meng S., Long L., Wu Z., Denisuk N., Yang Y., Wang L., Cao F., Zhu Y. (2020). Scalable dual-layer film with broadband infrared emission for sub-ambient daytime radiative cooling. Sol. Energy Mater. Sol. Cells.

[5] Wang X., Liu X., Li Z., Zhang H., Yang Z., Zhou H., Fan T. (2019). Scalable flexible hybrid membranes with photonic structures for daytime radiative cooling. Adv. Funct. Mater..

[6] Zhai Y., Ma Y., David S.N., Zhao D., Luo R., Tan G., Yang R., Yin X. (2017). Scalable-manufactured randomizedglass-polymer hybrid metamaterialfor daytime radiative cooling. Science.

[7] Huang Y., Pu M., Zhao Z., Li X., Ma X., Luo X. (2018). Broadband metamaterial as an “invisible” radiative cooling coat. Opt. Commun..

[8] Li W., Shi Y., Chen K., Zhu L., Fan S. (2017). A comprehensive photonic approach for solar cell cooling. ACS Photonics.

[9] Zhan Z., ElKabbash M., Li Z., Li X., Zhang J., Rutledge J., Singh S., Guo C. (2019). Enhancing thermoelectric output power via radiative cooling with nanoporous alumina. Nano Energy.

[10] He J., Zhang Q., Zhou Y., Chen Y., Ge H., Tang S. (2024). Bioinspired polymer films with surface ordered pyramid arrays and 3D hierarchical pores for enhanced passive radiative cooling. ACS Nano.

[11] Li X., Peoples J., Huang Z., Zhao Z., Qiu J., Ruan X. (2020). Full daytime sub-ambient radiative cooling in commercial-like paints with high figure of merit. Cell Rep. Phys. Sci..

[12] Zhang S., Cui C., Zhang F., Lu J., Su J., Han J. (2024). Flexible coated textile with remarkable passive daytime radiative cooling, UV resistance and hydrophobicity performance. Prog. Org. Coat..

[13] Zhang J., Xu S., Cai Y., Yi L. (2023). Colorfully coated cotton fabric for passive daytime radiative cooling. Prog. Org. Coat..

[14] Banik U., Agrawal A., Meddeb H., Sergeev O., Reininghaus N., Gotz-Kohler M., Gehrke K., Stuhrenberg J., Vehse M., Sznajder M. (2021). Efficient thin polymer coating as a selective thermal emitter for passive daytime radiative cooling. ACS Appl. Mater. Interfaces.

[15] Luo H., Yang M., Guo J., Zou W., Xu J., Zhao N. (2022). Hierarchical Porous Polymer Coatings Based on UV-Curing for Highly Efficient Passive All-Day Radiative Cooling. ACS Appl. Polym. Mater..

[16] An S., Shi B., Jiang M., Fu B., Song C., Tao P., Shang W., Deng T. (2023). Biological and bioinspired thermal energy regulation and utilization. Chem. Rev..

[17] Zhang H., Ly K.C.S., Liu X., Chen Z., Yan M., Wu Z., Wang X., Zheng Y., Zhou H., Fan T. (2020). Biologically inspired flexible photonic films for efficient passive radiative cooling. Proc. Natl. Acad. Sci. USA.

[18] Xie D., Yang Z., Liu X., Cui S., Zhou H., Fan T. (2019). Broadband omnidirectional light reflection and radiative heat dissipation in white beetles Goliathus goliatus. Soft Matter.

[19] Greiner A., Wendorff J.H. (2007). Electrospinning: A fascinating method for the preparation of ultrathin fibers. Angew. Chem.-Int. Ed..

[20] Miao D., Cheng N., Wang X., Yu J., Ding B. (2022). Integration of Janus wettability and heat conduction in hierarchically designed textiles for all-day personal radiative cooling. Nano Lett..

[21] Wu X.E., Wang Y., Liang X., Zhang Y., Bi P., Zhang M., Li S., Liang H., Wang S., Wang H. (2023). Durable radiative cooling multilayer silk textile with excellent comprehensive performance. Adv. Funct. Mater..

[22] He J., Zhang Q., Wu Y., Ju Y., Wang Y., Tang S. (2023). Scalable nanofibrous silk fibroin textile with excellent Mie scattering and high sweat evaporation ability for highly efficient passive personal thermal management. Chem. Eng. J..

[23] Cheng N., Miao D., Wang C., Lin Y., Babar A.A., Wang X., Wang Z., Yu J., Ding B. (2023). Nanosphere-structured hierarchically porous PVDF-HFP fabric for passive daytime radiative cooling via one-step water vapor-induced phase separation. Chem. Eng. J..

[24] Jaramillo-Fernandez J., Yang H., Schertel L., Whitworth G.L., Garcia P.D., Vignolini S., Sotomayor-Torres C.M. (2022). Highly-scattering cellulose-based films for radiative cooling. Adv. Sci..

[25] Zeng S., Pian S., Su M., Wang Z., Wu M., Liu X., Chen M., Xiang Y., Wu J., Zhang M. (2021). Hierarchical-morphology metafabric for scalable passive daytime radiative cooling. Science.

[26] Li D., Liu X., Li W., Lin Z., Zhu B., Li Z., Li J., Li B., Fan S., Xie J. (2021). Scalable and hierarchically designed polymer film as a selective thermal emitter for high-performance all-day radiative cooling. Nat. Nanotechnol..

[27] Zhang X., Cheng Z., Yang D., Dong Y., Shi X., Liang H., Wang F., Han H., Meng W., Shuai Y. (2023). Scalable bio-skin-inspired radiative cooling metafabric for breaking trade-off between optical properties and application requirements. ACS Photonics.

[28] Hsu P.C., Song A.Y., Catrysse P.B., Liu C., Peng Y., Xie J., Fan S., Cui Y. (2016). Radiative human body cooling by nanoporous polyethylene textile. Science.

[29] Zhao S., Da B., Peng F., Hu B., Gao C., Dai K., Zheng G., Liu C., Shen C. (2024). Facilely fabricated polyethylene film composed of directional microfibrils for passive radiative cooling. Polymer.

[30] Yao P., Chen Z., Liu T., Liao X., Yang Z., Li J., Jiang Y., Xu N., Li W., Zhu B. (2022). Spider-Silk-Inspired Nanocomposite Polymers for Durable Daytime Radiative Cooling. Adv. Mater..

[31] Yoshii F., Zhanshan Y., Isobe K., Shinozaki K., Makuuchi K. (1999). Electron beam crosslinked PEO and PEO/PVA hydrogels for wound dressing. Radiat. Phys. Chem..

[32] Jurkin T., Pucić I. (2013). Irradiation effects in poly(ethylene oxide)/silica nanocomposite films and gels. Polym. Eng. Sci..

[33] Kianfar P., Vitale A., Dalle Vacche S., Bongiovanni R. (2021). Enhancing properties and water resistance of PEO based electrospun nanofibrous membranes by photocrosslinking. J. Mater. Sci..

[34] Emami S.H., Salovey R. (2003). Crosslinked poly(ethylene oxide) hydrogels. J. Appl. Polym. Sci..

[35] Shekunov B.Y., Chattopadhyay P., Tong H.H., Chow A.H., Grossmann J.G. (2007). Structure and drug release in a crosslinked poly(ethylene oxide) hydrogel. J. Pharm. Sci..

[36] Xu F., Gough I., Dorogin J., Sheardown H., Hoare T. (2020). Nanostructured degradable macroporous hydrogel scaffolds with controllable internal morphologies via reactive electrospinning. Acta Biomater..

[37] Xue C., Wilson L.D. (2022). Preparation and characterization of salicylic acid grafted chitosan electrospun fibers. Carbohydr. Polym..

[38] Gao J., Guo H., Zhao L., Zhao X., Wang L. (2017). Water-stability and biological behavior of electrospun collagen/PEO fibers by environmental friendly crosslinking. Fibers Polym..

[39] Bonino C.A., Krebs M.D., Saquing C.D., Jeong S.I., Shearer K.L., Alsberg E., Khan S.A. (2011). Electrospinning alginate-based nanofibers: From blends to crosslinked low molecular weight alginate-only systems. Carbohydr. Polym..

[40] McAvoy K., Jones D., Thakur R.R.S. (2018). Synthesis and Characterisation ofPhotocrosslinked poly(ethylene glycol) diacrylate Implants for Sustained Ocular Drug Delivery. Pharm. Res..

[41] Lio G.E., Werlé J., Arduini M., Wiersma D.S., Manara J., Pattelli L. (2024). Radiative Cooling Potential of a Water-Based Paint Formulation under Realistic Application Conditions. ACS Appl. Opt. Mater..

[42] Li X., Pattelli L., Ding Z., Chen M., Zhao T., Li Y., Xu H., Pan L., Zhao J. (2024). A Novel BST@TPU Membrane with Superior UV Durability for Highly Efficient Daytime Radiative Cooling. Adv. Funct. Mater..

[43] Xu F., Zhang T., Xu Z., Zhao Y. (2024). Solid-solid phase change fibers with enhanced energy storage density for temperature management. J. Energy Storage.

[44] Jiang Y., Li B., Xu Z., Zhang T., Zhao Y. (2023). Emulsion-based monoliths with a solid-gel phase-transition and a solid–solid phase-transition for latent heat storage. J. Polymer Sci..

