# Supplementary material for: Water-Resistant Poly(ethylene oxide) Electrospun Membranes Enabled by In Situ UV-Cross-Linking for Efficient Daytime Radiative Cooling
Source: Molecules. 2025 Jan 20;30(2):421. doi: 10.3390/molecules30020421 (PMC11767364; doi:10.3390/molecules30020421)
Supplement: Supplementary file 1 [file molecules-30-00421-s001.zip › molecules-3443255-supplementary.pdf]

Supporting Information

Water-resistant poly(ethylene oxide) electrospun membranes enabled by in situ UV-crosslinking for efficient daytime radiative cooling

Haiyan Zhang,[#,a] Qingpeng Wang,[#,a] Zhiguang Xu,[*,b] Yan Zhao[*,a]

[a] College of Textile and Clothing Engineering, Soochow University, Suzhou 215123, China

[b] College of Biological, Chemical Sciences and Engineering, China-Australia Institute for Advanced Materials and Manufacturing, Jiaxing University, Jiaxing 314001, China

[#]These authors contribute equally.

*Corresponding authors.

E-mail addresses: zhiguang.xu@zjxu.edu.cn (Z. Xu), yanzhao@suda.edu.cn (Y. Zhao).

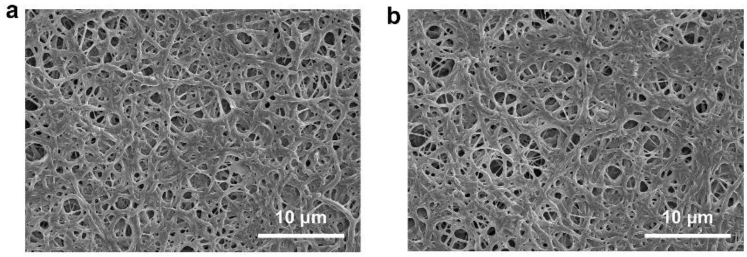

Figure S1. SEM images of PEO-ISA/Al_2O_3-T10 after (a) the second and (b) the third water soaking-drying cycle (water soaking time is 1 h).

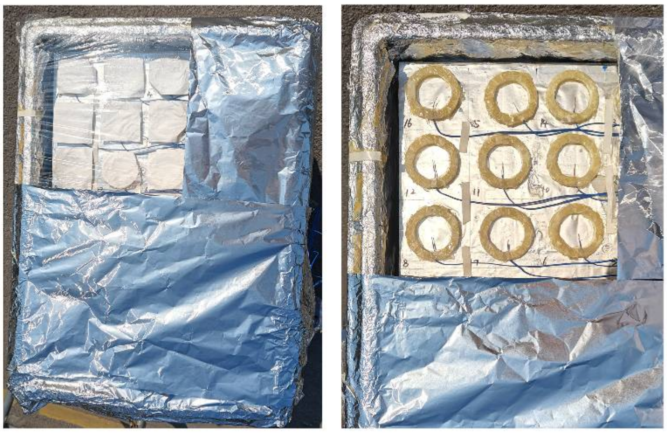

Figure S2. Digital photos of the test setup using for outdoor cooling performance test: with samples (left), and without samples (right, showing the position of the thermocouples).

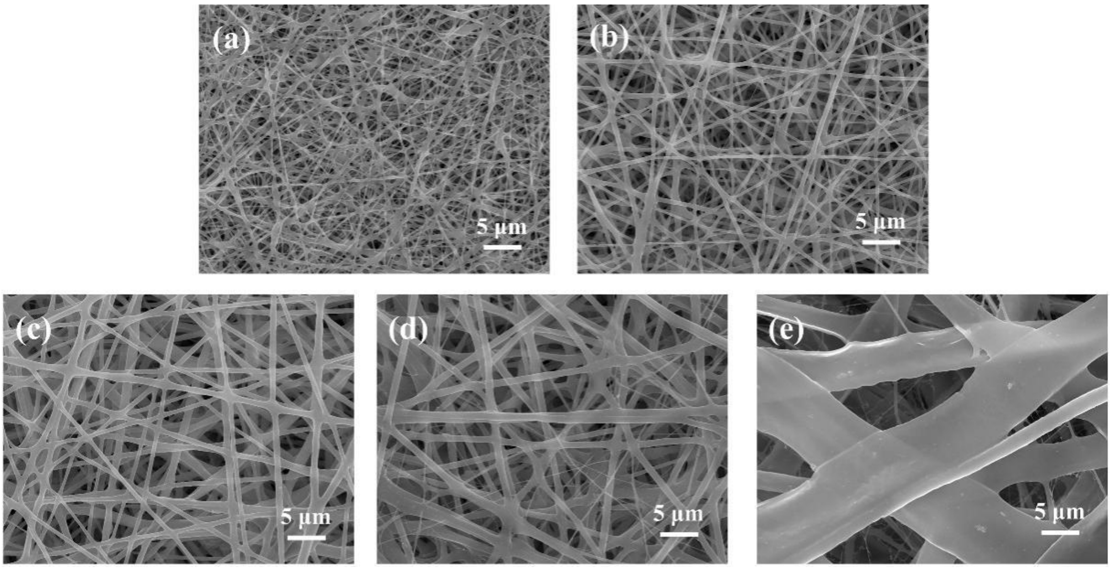

Figure S3. SEM images of PEO electrospun membranes prepared with spinning solutions having different concentrations: (a) 3 wt%; (b) 4 wt%; (c) 5 wt%; (d) 6 wt%; (e) 7 wt%.

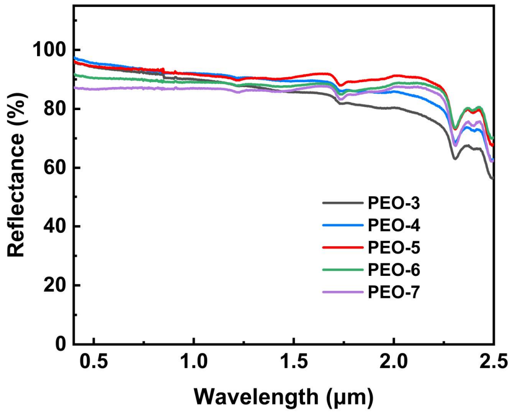

Figure S4. Reflectance of PEO electrospun membranes prepared with spinning solutions having different concentrations.

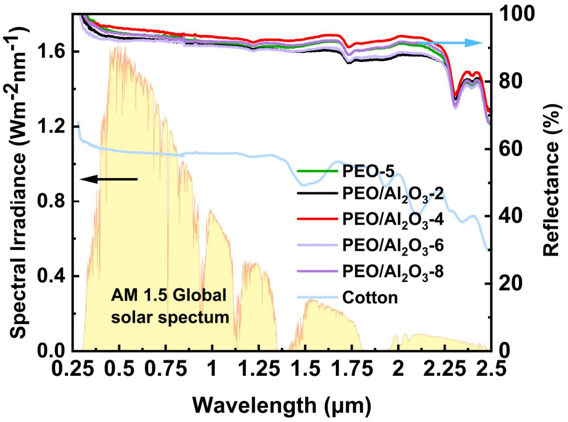

Figure S5. Reflectance of PEO/Al_2O_3 electrospun membranes prepared with different Al_2O_3 mass fractions.

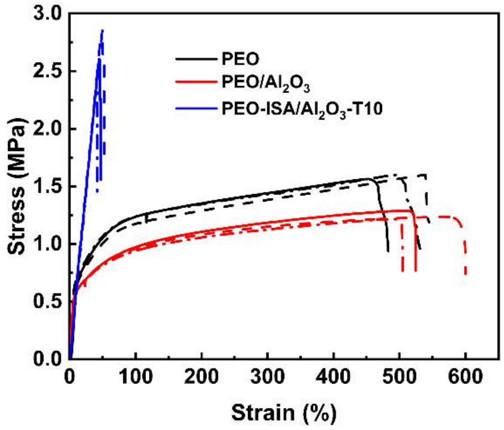

Figure S6. Tensile stress-strain curves for the PEO, PEO/Al_2O_3, and $PEO\text{-}ISA/Al_2O_3\text{-}T10$ electrospun membranes.
